# Unilateral Thyroid Hypoplasia in a Euthyroid Adult: A Rare Congenital Anomaly

**DOI:** 10.7759/cureus.94437

**Published:** 2025-10-13

**Authors:** Neha Butt, Atba Ausama Khan, Daayl N Mirza

**Affiliations:** 1 Internal Medicine, Ziauddin University, Karachi, PAK

**Keywords:** congenital birth defect, thyroid hypoplasia, thyroid pathology, thyroid scan, ultrasound (us)

## Abstract

Thyroid hypoplasia is described as a rare congenital anomaly. It is characterized by incomplete development of thyroid tissue, which can be differentiated from thyroid hemiagenesis, where a lobe is completely absent. These cases mostly present asymptomatically and are often found when imaged for unrelated conditions.

In this report, we present a case of a 22-year-old euthyroid female who was incidentally discovered to have a hypoplastic left thyroid lobe during ultrasonography. After undergoing further investigations, the thyroid functioning tests were within a normal range (thyroid-stimulating hormone (TSH) 0.751 mIU/L, triiodothyronine (T3) 1.99 pg/mL, thyroxine (T4) 8.21 µg/dL). In addition, an isotope thyroid scan was done, confirming no functioning tissue in the hypoplastic lobe.

This case study underlines the significance of differentiating between thyroid hemiagenesis and hypoplasia. In addition, it also highlights the importance of long-term follow-up for assessment of associated thyroid pathologies.

## Introduction

Congenital anomalies associated with the thyroid gland are rare, with an estimated prevalence of 1 in 2000 to 4000 individuals worldwide. They include agenesis, hypoplasia, and ectopy, which, depending on the extent of glandular development, may result in variable thyroid hormone dysfunction. Thyroid hemiagenesis is defined as the complete absence of one lobe with or without the isthmus. In comparison, thyroid hypoplasia refers to a small, underdeveloped lobe that may or may not be functional. Although both anomalies have been described globally, data from the South Asian population remains limited [1,2].

Most cases of both conditions are discovered incidentally during imaging performed for unrelated problems rather than during evaluation for thyroid disease [1-3]. Both anomalies can coexist with other thyroid disorders, including nodules, autoimmune thyroiditis, Graves’ disease, and Hashimoto’s thyroiditis [4-6].

We present a case of unilateral thyroid hypoplasia in a young female, incidentally discovered during an ultrasound performed for a posterior cervical swelling. This highlights the need to maintain a high index of suspicion during neck imaging, even when thyroid disease is not clinically suspected, and contributes region-specific data to the limited literature on congenital thyroid anomalies.

## Case presentation

**Figure 1:**
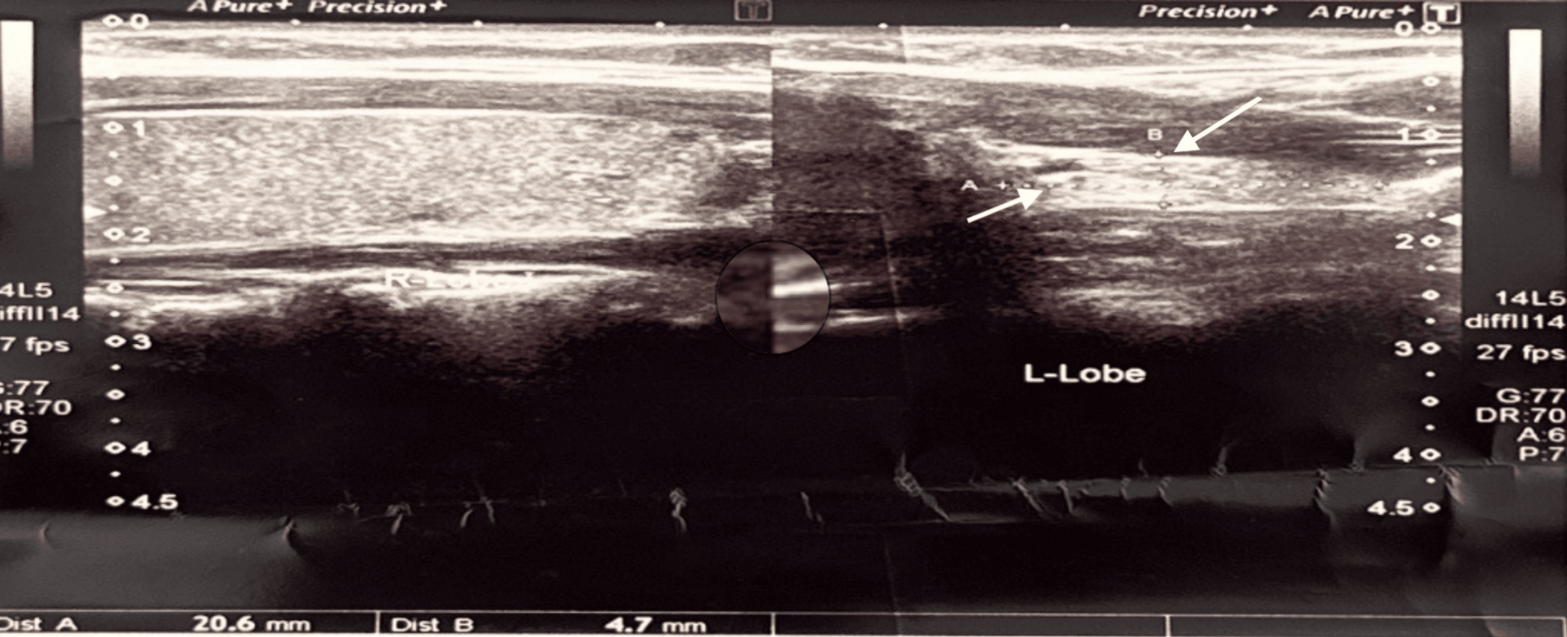
Thyroid ultrasound of a 22-year-old female showing the size of the left lobe of the thyroid, measuring 2.0 × 0.47 cm.

**Figure 2:**
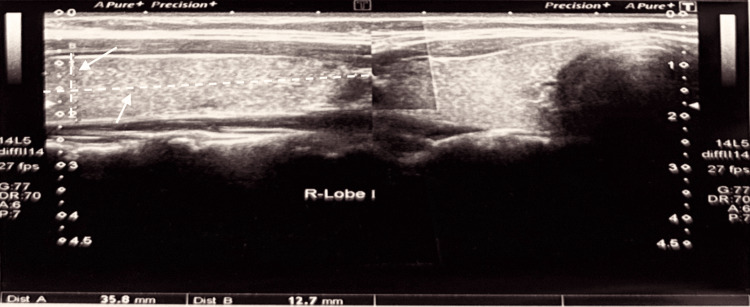
Thyroid ultrasound of a 22-year-old female showing the size of the right lobe of the thyroid, measuring 3.5 × 1.2 cm.

**Figure 3:**
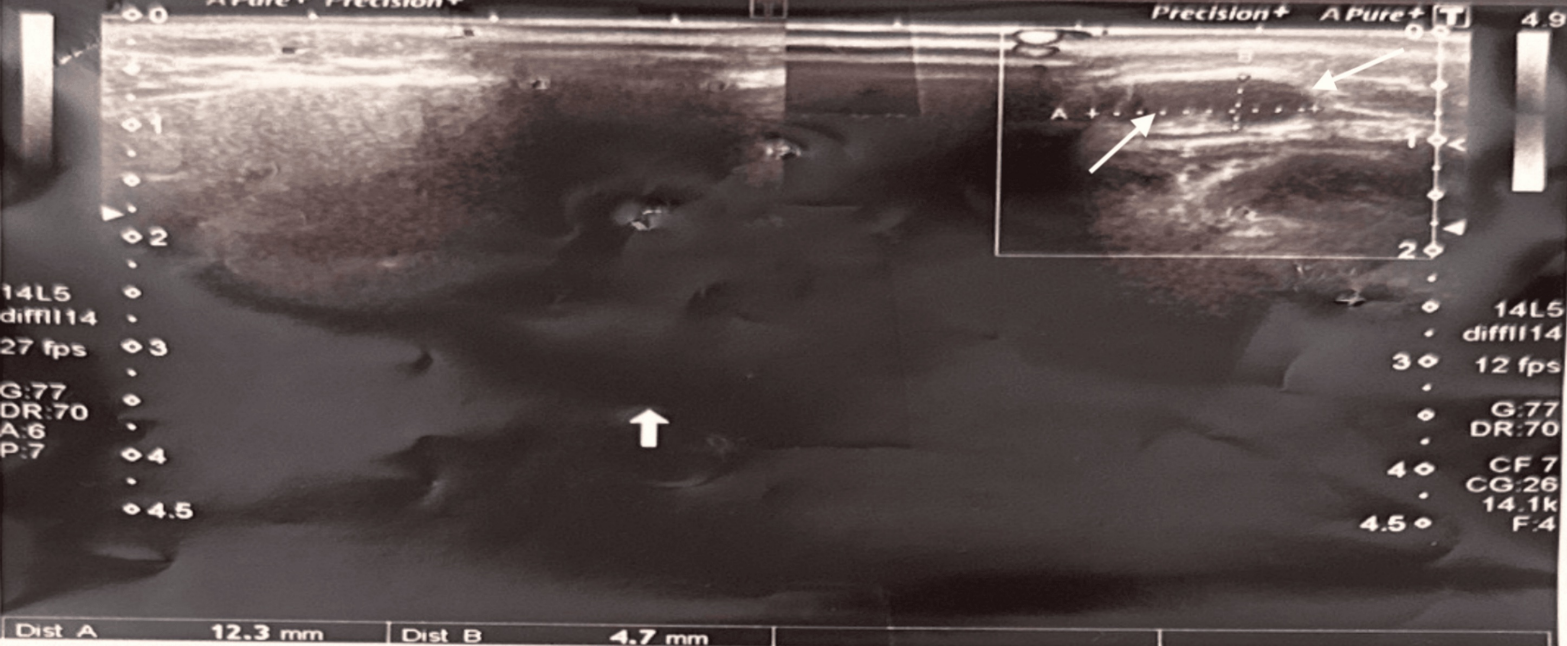
Ultrasound scan of a 22-year-old female showing a nuchal lymph node measuring 1.23 × 0.47 cm.

A 22-year-old female presented after an incidental finding on neck ultrasonography performed for a posterior cervical swelling. The scan confirmed a markedly hypoplastic left lobe (2.0 × 0.47 cm) and a normal right lobe (3.5 × 1.2 cm), with preserved isthmus (Figures 1-2). No calcifications, masses, or cysts were detected. Bilateral benign-appearing cervical lymph nodes were seen (the largest measuring 9.4 × 2.6 mm on the left). A nuchal lymph node (1.23 × 0.47 cm) appeared inflamed but reactive (Figure 3).

When the patient was clinically assessed, she was asymptomatic and euthyroid. Laboratory results showed normal thyroid function (thyroid-stimulating hormone (TSH) 0.751 mIU/L, triiodothyronine (T3) 1.99 pg/mL, and thyroxine (T4) 8.21 µg/dL) (Table 1).

**Table 1:** Thyroid function test results. TSH: thyroid-stimulating hormone; T3: triiodothyronine; T4: thyroxine

Test	Laboratory Value	Unit	Reference Ranges
Serum TSH	0.751	µIU/mL	0.4-4.2
Serum T4	8.21	µg/dL	5.5-11
Serum T3	1.99	nmol/L	1.08-3.14

A Tc-99m pertechnetate scan confirmed uptake in the right lobe only, with no functioning tissue within the hypoplastic left lobe. A small pyramidal lobe was also noted. The patient was counselled about possible symptoms of thyroid dysfunction and advised regular follow-up, especially during pregnancy.

## Discussion

Differentiating hypoplasia from hemiagenesis is clinically important. In thyroid hemiagensis, a lobe is completely absent, whereas in hypoplasia, remnant tissue exists but is markedly small and often nonfunctional [1,2]. Our case demonstrates the latter.

It is vital to differentiate the two as it has practical implications for patient management and prognosis. In hypoplasia, residual nonfunctional tissue may develop autoimmune thyroiditis or nodular changes over time, which necessitates long-term biochemical and imaging surveillance. On the other hand, in hemiagenesis, compensatory hypertrophy or hyperfunction of the contralateral lobe can occur [1,2]. Failure to recognize this distinction may lead to misdiagnosis of lobar atrophy, unnecessary surgical intervention, or missed follow-up for potential thyroid dysfunction.

The exact prevalence of thyroid hypoplasia is unknown, partly due to underdiagnosis. Thyroid hypoplasia (THA) is estimated at 0.02-0.05% in large population studies [7,8], but reports of unilateral hypoplasia are even rarer. Females are more frequently affected, and the left lobe is most often involved [7]. Both anomalies result from abnormal development of the thyroid diverticulum during the fifth week of gestation [9]. Incomplete migration or growth arrest may lead to hypoplasia rather than agenesis. Most patients remain asymptomatic, but associated conditions such as nodules, autoimmune thyroiditis, or compensatory hypertrophy of the contralateral lobe can occur [10-12]. Ultrasonography is the first-line imaging modality, while scintigraphy is useful to confirm absent or non-functioning tissue.

Several international case reports and series highlight the rarity of structural thyroid anomalies and their variable clinical presentation. Peña et al. described an incidental finding of thyroid hemiagenesis in a 55-year-old woman with normal thyroid function, reflecting that such anomalies may remain asymptomatic until imaging for unrelated causes [5]. Similarly, Wu et al. presented a small series of thyroid hemiagenesis cases, reinforcing the spectrum of laterality, functional status, and associated thyroid pathology [13]. Additionally, Stoupa et al. reported neonatal cases of thyroid hypoplasia associated with TPO gene mutations, illustrating how similar developmental anomalies may present at different ages and with diverse functional outcomes [14]. In contrast, our case represents an adult, euthyroid patient with unilateral hypoplasia identified incidentally, further emphasizing the diagnostic variability and benign course such anomalies may follow.

In our patient, the anomaly was identified incidentally on neck ultrasonography performed for evaluation of a posterior cervical swelling, underscoring the value of imaging in revealing asymptomatic congenital thyroid anomalies. There is no consensus on management, but regular follow-up is advisable due to the increased risk of thyroid dysfunction and nodular disease [12-14].

## Conclusions

This case demonstrates unilateral thyroid hypoplasia in a euthyroid adult. Unlike hemiagenesis, a hypoplastic lobe is present but underdeveloped and nonfunctional. It is significant to differentiate between thyroid hemiagenesis and unilateral hypoplasia as it guides prognosis, follow-up strategy, and anticipatory counseling. By accurate distinction, there is prevention of unnecessary interventions while supporting risk-based surveillance for thyroid dysfunction or nodular disease. Comparison with international reports reinforces that such anomalies may manifest across ages and functional states, underscoring the importance of awareness and appropriate long-term monitoring. While patients may remain asymptomatic, counseling and long-term follow-up are important due to the risk of autoimmune thyroid disease and neoplasia.
